# A case of sternal osteomyelitis during treatment with everolimus for recurrent breast cancer

**DOI:** 10.1186/s40792-022-01376-y

**Published:** 2022-01-28

**Authors:** Kaori Abe, Masafumi Shimoda, Tetsuhiro Yoshinami, Yoshiaki Sota, Tomohiro Miyake, Tomonori Tanei, Naofumi Kagara, Yasuto Naoi, Kenzo Shimazu

**Affiliations:** Department of Breast and Endocrine Surgery, Osaka University Graduate School of Medicine, 2-2-E10 Yamadaoka, Suita, Osaka 565-0871 Japan

**Keywords:** Sternal osteomyelitis, Everolimus, mTOR inhibitor, Breast cancer, Denosumab

## Abstract

**Background:**

Everolimus is a mechanistic-target-of-rapamycin (mTOR) inhibitor bearing a potent antitumor effect against hormone receptor-positive breast cancer. Here, we report the case of a patient with recurrent breast cancer who developed osteomyelitis during the treatment with everolimus plus exemestane.

**Case presentation:**

A 56-year-old woman with early-stage breast cancer underwent right mastectomy and axillary lymph node dissection at the age of 45. Four years after the surgery, she experienced relapse at the chest wall. Radiotherapy was performed on the chest wall, including the sternum, and denosumab was administered. After several regimens of hormonal therapies, everolimus in combination with exemestane was administered. Three months later, the patient visited our clinic because of continuous fever. A computed tomography scan showed an osteolytic change in the sternal bone with pneumomediastinum, which indicated sternal osteomyelitis. Extensive debridement followed by secondary reconstruction of the chest wall was successfully performed.

**Conclusions:**

Everolimus may cause osteomyelitis of the affected bone as a result of tumor necrosis. Everolimus-induced osteomyelitis may be manageable by extensive debridement performed without delay.

## Background

Breast cancer is the most common cancer in women, threatening women’s health worldwide [1]. Approximately 70% of breast cancers are positive for hormone receptors (HRs), including the estrogen receptor (ER) and the progesterone receptor (PR). Hormonal therapy with the use of anti-estrogen drugs or aromatase inhibitors is effective for patients with early and advanced HR-positive breast cancer [2]. In recent years, patients with HR-positive advanced breast cancer have been treated with molecular-targeted therapies, including cyclin-dependent kinase 4/6 inhibitors, phosphoinositide-3 kinase (PI3K) inhibitors, and a mechanistic-target-of-rapamycin (mTOR) inhibitor, concurrently with hormonal therapy to augment the effect of the hormonal therapy.

Everolimus is an mTOR inhibitor used in combination with the aromatase inhibitor exemestane for postmenopausal women with HR-positive advanced breast cancer [3]. mTOR is a serine/threonine kinase that regulates fundamental physiological processes such as cell proliferation, cell motility, cell metabolism, and neoangiogenesis. mTOR is a downstream target of the PI3K-protein kinase B (AKT) pathway, which is often reactivated in HR-positive breast cancer cells, resulting in resistance to hormonal therapy [3]. Despite the high efficacy of everolimus for treating HR-positive advanced breast cancer, it can result in serious adverse effects, including interstitial pneumonia, infections, stomatitis, delayed wound healing, and hyperglycemia, because mTOR inhibition suppresses basic cellular processes. Here, we present the case of a patient with sternal osteomyelitis that emerged during the administration of everolimus plus exemestane and conclude with a review of the literature.

## Case presentation

**Fig. 1:**
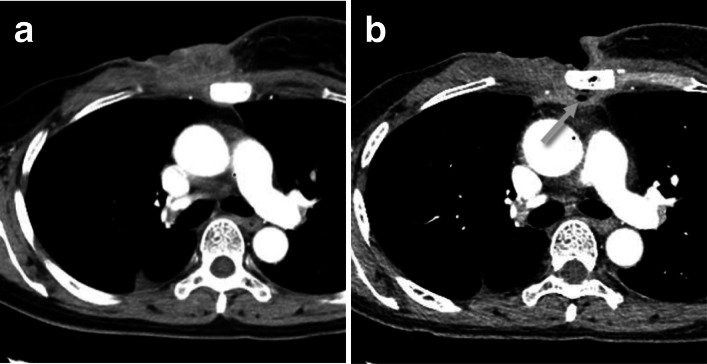
CT findings of the tumor in the chest wall. **a** A CT image obtained before the administration of everolimus plus exemestane. **b** A CT image obtained when the patient experienced fever during treatment with everolimus and exemestane. An arrow indicates an air bubble

**Fig. 2:**
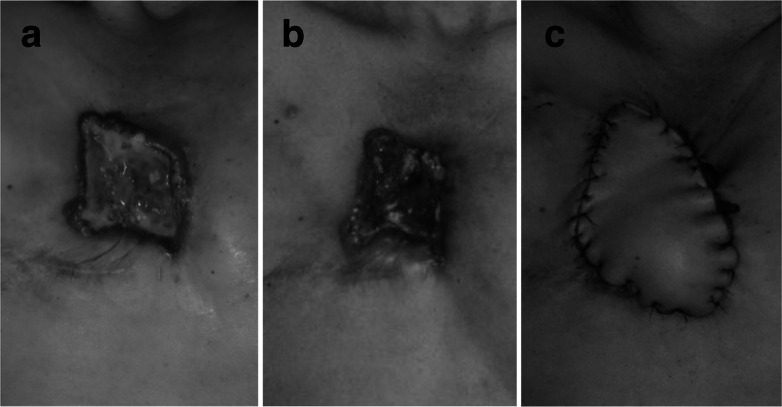
Photographs of the affected chest wall. Photographs were taken before debridement of the necrotic tissues (**a**), after debridement (**b**), and after reconstruction using a latissimus dorsi myocutaneous flap (**c**)

A woman with right breast cancer underwent right mastectomy and axillary lymph node dissection at the age of 45 at a municipal hospital. The patient had no other complications and was not a smoker. The pathological findings indicated that the tumor was invasive ductal carcinoma with Bloom–Richardson grade 3, and the TNM classification was pT2N1M0. Immunohistochemical examinations revealed that the tumor was ER-positive, PR-positive and human epidermal growth factor receptor type 2 (HER2)-negative. After the surgery, the patient received adjuvant hormonal therapy, including tamoxifen and goserelin. Three years and nine months after the surgery, the breast carcinoma recurred in the supraclavicular lymph nodes. Letrozole and goserelin were administered, and the patient was referred to our hospital 4 years and 5 months after the surgery. At that time, the breast carcinoma had metastasized to the sternum, the right parasternal lymph nodes, and the right mediastinal lymph nodes. Radiotherapy was performed on the affected sternum, superior mediastinum, and right supraclavicular fossa at a dose of 60 Gy in 30 daily fractions. The patient was subsequently treated in sequence with leuprorelin and exemestane, fulvestrant, capecitabine, palbociclib and fulvestrant, but the tumor in the chest wall eventually formed a large mass with a skin ulcer (Fig. 1a). Everolimus and exemestane were administered to the patient at the age of 56 years, 10 years and 6 months after the initial surgery. Three months later, the tumor size remained the same as the baseline, and serum CA15-3 levels decreased from 140.8 U/mL before everolimus administration to 53.3 U/mL. However, in this period, the ulcer reached the sternum, and the patient’s body temperature rose to 38.4 °C. Ciprofloxacin did not alleviate the symptoms. Computed tomography (CT) revealed osteolysis of the sternum with air bubbles in the anterior mediastinum, suggesting the presence of sternal osteomyelitis (Fig. 1b). The wound was infected with methicillin-resistant *Staphylococcus epidermidis* and multiple anaerobes, so vancomycin and meropenem were administrated. Due to the large defects in the skin and soft tissues, a two-step surgical intervention was planned. In the first step, 40 days after the symptoms began, the necrotic tissues were removed. Twelve days after debridement, reconstruction of the chest wall was performed using a latissimus dorsi myocutaneous flap. The postoperative course was uneventful, and flap engraftment was satisfactory (Fig. 2).

When histological observation was conducted, it was noticed that most of the tumor nests had been replaced by necrotic tissues, although a few of the tumor cells were still viable. The surgical margin of the skin was clear, but the deep stump was positive, indicating that the chest wall tumor persisted. Thus, paclitaxel administration was initiated 11 days after the reconstruction. The patient was doing well without the recurrence of sternal osteomyelitis 1 year and 8 months after the reconstruction.

## Discussion

Osteomyelitis is a disease caused by bone infection, which is a rare but serious condition. Common causes of osteomyelitis include bone injury, bone or joint surgery, and lower limb infections due to diabetes [4]. Osteomyelitis secondary to breast cancer is extremely rare, except for osteonecrosis of the jaw induced by bone-modifying agents. In this case presentation, the patient’s osteomyelitis was caused by exposure of the sternum, presumably related to the treatments the patient had been receiving.

The generation of skin ulcers reaching the sternum may have resulted from disease progression or the treatments. However, it was unlikely that the progression of the disease caused the ulcer formation as no new lesions were observed during the treatment with exemestane and everolimus and the tumor marker CA15-3 levels were diminished as well, although tumor markers do not reflect the tumor burden for all instances. Rather, the pathological findings of the surgical specimen indicated that exemestane and everolimus administration might have been effective, resulting in necrosis of the tumor in the chest wall. Necrotic tissue is usually absorbed and replaced by granulated tissue, leaving no defects in the necrotic area. However, the defect remained open in this case. This possibly resulted from the pharmacological effects of everolimus, including delayed wound healing, inhibition of neoangiogenesis, and immunosuppression.

Everolimus inhibits mTOR, leading to the inhibition of cell proliferation, cell motility, cell metabolism, and neoangiogenesis [5]. Moreover, as mTOR is reactivated by the stimulatory cytokine interleukin-2 through the PI3K–AKT pathway in immune cells, mTOR inhibition by everolimus leads to the suppression of immune cells such as T cells, dendritic cells, and natural killer cells [6]. Although reports on the formation of tissue defects with everolimus are limited, drugs bearing pharmacological effects similar to everolimus often cause tissue defects. For example, bevacizumab, a monoclonal antibody targeting vascular endothelial growth factor (VEGF)-A, inhibits tumor neoangiogenesis, thereby delaying wound healing. Bevacizumab administration in patients with skin metastasis of breast cancer often results in extensive and persistent skin necrosis [7]. Everolimus may also inhibit the expression of VEGF through the downregulation of the expression of HIF-1α, which is a transcriptional target of mTOR [8, 9]. Another example is lenvatinib, a multikinase inhibitor used for patients with thyroid, renal, and liver cancer. Lenvatinib inhibits VEGF receptors 1–3, platelet-derived growth factor receptor-α, fibroblast growth factor receptors (FGFR) 1–4, KIT, and RET, among which inhibition of VEGF receptor 2 contributes to the major pharmacological effect, including the inhibition of tumor neoangiogenesis. Consequently, lenvatinib also delays wound healing [10]. Fistula formation as a result of tumor necrosis in the neck often results in severe conditions, including blood vessel rupture and death [11]. Thus, it should be noted that in patients with cancerous skin ulcers, drugs that can cause delayed wound healing, such as everolimus, may result in extensive skin necrosis.

Additionally, the radiotherapy of the affected chest wall performed 6 years before the onset of osteolysis and the concurrent administration of denosumab might have increased the risk of tissue defects, which had been observed in the present case. Radiotherapy obviously impairs wound healing by extensively affecting cells [12]. Denosumab, a monoclonal antibody against receptor activator of nuclear factor κB ligand, often causes osteonecrosis of the jaw, which is promoted by bone infection [13]. The etiology of osteonecrosis of the jaw induced by denosumab is similar to the present case in which sternal bone infection was observed.

Sternal osteomyelitis has a mortality rate of 31% when surgery is not performed at the right time because sternal osteomyelitis requires early debridement instead of conservative treatment [14]. Although criteria for the appropriate resection area in osteomyelitis have not been determined, extensive resection of the affected area followed by appropriate skin flap construction may be necessary for an early recovery, as seen in the present case.

## Conclusions

Similar to drugs that cause delayed wound healing, everolimus may cause osteomyelitis of the affected bone as a result of tumor necrosis. The careful use of everolimus may be necessary in patients who bear cancerous skin ulcers. Additionally, extensive debridement without delay may be necessary for the management of osteomyelitis caused by tumor necrosis.

## Data Availability

The dataset supporting the conclusion of this article is included within the article.

## Abbreviations

AKT: Protein kinase B
CT: Computed tomography
ER: Estrogen receptor
FGFR: Fibroblast growth factor receptors
HER2: Human epidermal growth factor receptor type 2
HR: Hormone receptor
mTOR: Mechanistic target of rapamycin
PR: Progesterone receptor
PI3K: Phosphoinositide-3 kinase
VEGF: Vascular endothelial growth factor
